# Tryptophan metabolism in pancreatic cancer: A review

**DOI:** 10.1097/MD.0000000000044904

**Published:** 2025-10-10

**Authors:** Yifei Gao, Lei Yang, Ximo Wang

**Affiliations:** a Artificial Cell Engineering Technology Research Center, Tianjin Institute of Hepatobiliary Disease, Tianjin Medical University Third Center Clinical College, Tianjin Third Central Hospital, Tianjin, China; b Tianjin Key Laboratory of Acute Abdomen Disease Associated Organ Injury and ITCWM Repair, Institute of Integrative Medicine for Acute Abdominal Diseases, Tianjin, China.

**Keywords:** gut microbiota, immune microenvironment, pancreatic cancer, tryptophan metabolism

## Abstract

Our study provides a detailed exploration of the role of tryptophan metabolism in pancreatic cancer, including its normal function, the impact of targeted drugs, and the influence of gut microbiota on metabolism. Additionally, the article discusses how tryptophan metabolism affects early diagnosis, immune microenvironment, metastasis and proliferation, and pharmaceutical interventions in pancreatic cancer. Research has found that tryptophan and its metabolites can serve as potential biomarkers for early diagnosis and may improve the prognosis of pancreatic cancer patients by modulating the tumor immune microenvironment.

## 1. Introduction

Pancreatic ductal adenocarcinoma (PDAC) is the deadliest tumor of the digestive system, responsible for 90% of pancreatic cancer (PC) cases. The survival rate for PC over 5 years is meager as early diagnosis is difficult to detect.^[1]^ 50% of patients are already in the metastatic stage upon discovery, and the effectiveness of immunotherapy is poor.^[2]^ Tryptophan (Trp) is a crucial amino acid that humans can solely acquire through dietary intake. As an essential component of proteins, Trp can coordinate the body’s response to the external environment and dietary intake. Additionally, Trp serves as a precursor to many vital physiological metabolites, with its metabolites playing a crucial role as signaling molecules in the growth and regulation of PC cells. There has been a growing interest in the involvement of Trp metabolism in the progression of PC. This article comprehensively reviews the effects of Trp and its metabolites on PC, exploring the potential links between Trp metabolism and the diagnosis and treatment of the disease.

## 2. Influences on tryptophan metabolism

### 2.1. Normal functions of tryptophan metabolism

**Figure 1.**
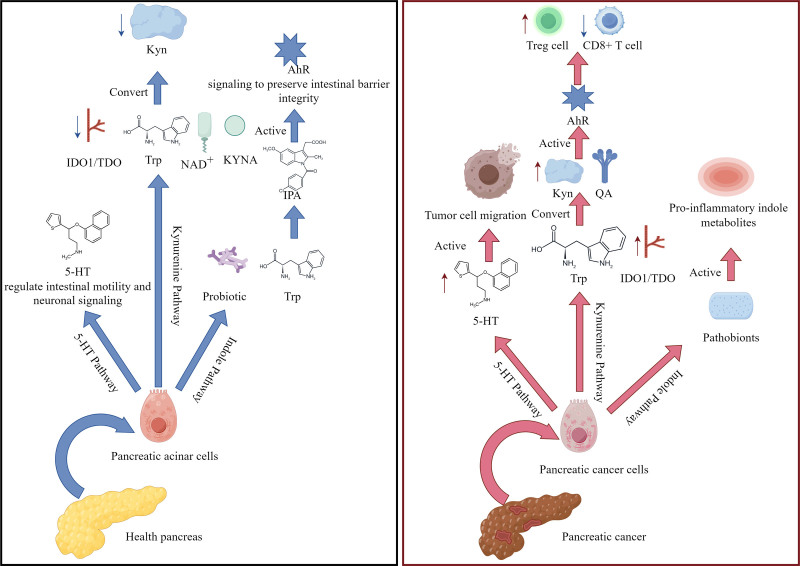
Trytophan metabolism under normal healthy condition vs Trytophan metabolism during pancreatic cancer. AhR = aryl hydrocarbon receptor, 5HT = serotonin, KYN = kynurenine, KYNA = kynurenic acid, IDO1 = indoleamine 2,3-dioxygenase 1, TDO2 = tryptophan 2,3-dioxygenase, Trp = Tryptophan.

Trp regulates various physiological functions such as sleep, mood, and immune response through multiple metabolic pathways. The human metabolizes Trp through 4 ways. Firstly, intestinal epithelial cells transport Trp through the apical membrane to the intestinal stroma and mesentery after ingesting protein. Secondly, Trp enters the liver, where most of it is oxidized to acetyl coenzyme A for synthesizing nicotinamide adenine dinucleotide (NAD^+^). Thirdly, under inflammatory stimulation, kynurenine (KYN) metabolites are released by myeloid cells, inhibiting T cell response. Lastly, astrocytes, microglia, and neurons absorb Trp, KYN, and 3-hydroxykynurenine through the blood-brain barrier. Kynurenic acid (KYNA), a neuroprotective substance, is produced by astrocytes, while microglia have quinolinic acid (QA) and 5-hydroxytryptamine, which are neurotoxic KYN metabolites.^[3]^ As summarized in Figure 1, Approximately 90% of Trp is metabolized through the KYN pathway in the liver under normal conditions, whereas in PC, this pathway is dysregulated with upregulated indoleamine 2,3-dioxygenase 1 (IDO1) activity and accumulation of immunosuppressive KYN derivatives. Notably, the elevated IDO1 activity in PC not only drives immunosuppression through KYN metabolites but also impedes dendritic cell infiltration into tumor tissues, further exacerbating immune evasion.^[4]^

### 2.2. The impact of targeted drugs on tryptophan metabolism

Trp is metabolized through the KYN pathway to generate KYN, QA, and coenzyme NAD^+^. The initial step of this process is catalyzed by tryptophan 2,3-dioxygenase (TDO2), indoleamine 2,3-dioxygenase 2, and indoleamine 2,3-dioxygenase 1. In the typical metabolism process, the enzyme IDO1 is responsible for breaking down Trp into KYN within the liver or other non-liver cells. Any leftover Trp is metabolized by bacteria or transformed into 5-hydroxytryptamine or serotonin (5-HT). Bacteria, on the other hand, convert Trp into indole compounds. Combining these indole compounds with dietary indole compounds, as well as IDO1 and KYN, provides ligands for the transcription factor aryl hydrocarbon receptor (AhR). In the metabolic environment of PC, IDO1 is overexpressed, leading to Trp depletion, elevated levels of L-KYN, sustained AhR activation, and potentially more robust AhR activation and excessive production of IL-22/IL-17, as well as excessive cell proliferation. When this occurs, it triggers the activation of regulatory T cells (Treg), which create an environment that is immune-tolerant and supports the growth of tumors.^[5]^ Various types of cancer cells, such as breast malignancy, cervical malignancy, and brain malignancy, show increased levels of IDO1 and TDO2. These elevated levels are linked to the invasiveness of the tumor and a negative prognosis for patients, as indicated by research.^[6,7]^ Trp depletion in tumor microenvironment suppresses the immune system, promoting the immune escape of cancer cells by producing KNY and other metabolites.^[8–10]^ Inhibitors of these enzymes have emerged as a novel approach for cancer immunotherapy. In addition, with the increasing research in traditional Chinese medicine, there have been many discoveries in developing targeted drugs. Analysis of serum metabolomics has revealed that the utilization of polysaccharides derived from Shenling Baizhu San substantially enhances Trp metabolism, mainly by stimulating the KYN pathway that triggers the expression of AhR and cytochrome P450 family 1 subfamily A member 1 (CYP450 1A1). This process also promotes the expression of IL-10 in the colon.^[11]^

### 2.3. The impact of intestinal microbiota on the metabolism of tryptophan

Intestinal microorganisms can participate in the regulation of Trp metabolism by directly metabolizing Trp or affecting host metabolic pathways, thereby affecting the immune balance and disease status of the body. The digestive system contains a variety of helpful bacteria, such as Bacteroides, Clostridium, Clostridium, Bifidobacterium, Lactobacillus, and Ruminococcus.^[12]^ These bacteria convert small amounts of Trp into indole and its derivatives, including indole-3-propionic acid, indole-3-acetic acid, and indole acrylic acid.^[13]^ For instance, lactobacilli change Trp into indole-3-aldehyde, whereas Pseudomonas, Staphylococcus, and Providencia promote the conversion of Trp into indole-3-acetic acid.^[14]^

## 3. The impact of tryptophan metabolism on pancreatic cancer

### 3.1. The value of tryptophan and its metabolites in early diagnosis of pancreatic cancer

The changes in Trp metabolism products show a distinct pattern in PC patients, indicating potential biomarkers for early diagnosis. In 2015, Fukutake conducted a study collecting samples from 360 PC patients, 28 chronic pancreatitis patients, and 8372 healthy individuals. They utilized liquid chromatography-mass spectrometry to examine the samples and discovered that the plasma levels of Trp and histidine were notably reduced in patients with PC. This suggests that measuring the plasma free amino acid profile, especially the plasma Trp index, could be useful in diagnosing asymptomatic and resectable PC patients at an early stage.^[15]^ In 2020, Xiong expanded the use of liquid chromatography in combination with high-resolution mass spectrometry to thoroughly examine the variations in serum metabolism among PC patients, healthy individuals, and patients with benign diseases. They used the continuous window acquisition of all theoretical mass spectra for relative quantification to reveal that the Trp differences between PC patients and healthy individuals may provide sensitive blood-borne diagnostic markers for the presence of PC or its precursor lesions.^[16]^

Furthermore, gut microbiota and its metabolites can be used for early detection of pancreatic malignancy in mouse models. After detecting the abundance of gut microbiota in mice, it has been found that the progression of PC is closely related to polyamines. Clinical studies have also revealed an increase in polyamine concentrations in serum samples of PC patients. Therefore, polyamines may function as potential early indicators for detecting pancreatic malignancy. The study also found that specific lactobacilli were detected as the tumor progressed in mice (samples at 4 months), which were not found in earlier age groups, indicating the association of specific lactobacilli with polyamine metabolism.^[17]^ Notably, recent studies further highlight the translational potential of microbial metabolites in cancer diagnostics. In 2024, Zhang systematically reviewed the role of microbiota-derived tryptophan metabolites (e.g., indole derivatives and polyamines) as biomarkers across multiple endocrine tumors, including pancreatic neuroendocrine neoplasms, and demonstrated their capacity to distinguish malignant lesions from benign conditions through multi-omics profiling.^[18]^ In conclusion, numerous studies suggest the significant value of Trp metabolism products for early PC diagnosis.

### 3.2. The influence of tryptophan metabolites on the tumor immune microenvironment

**Figure 2.**
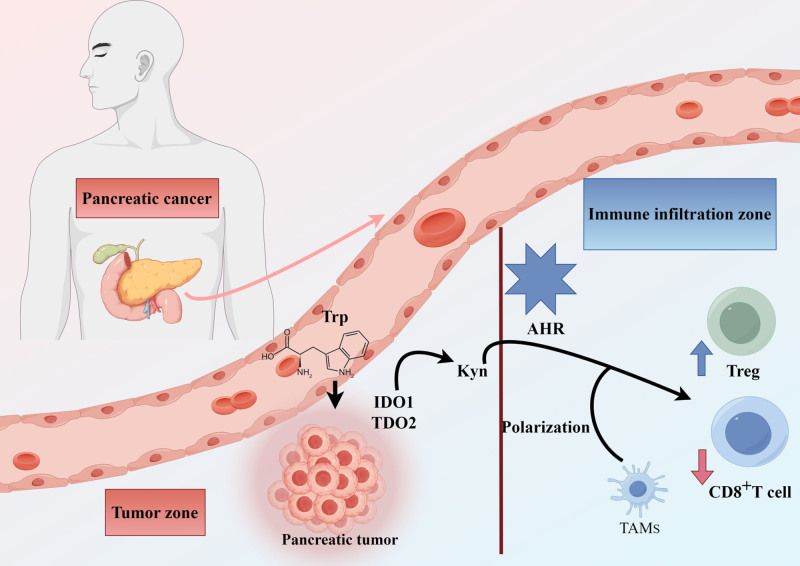
Immunosuppressive mechanism of pancreatic cancer mediated by tryptophan catabolism. AhR = aryl hydrocarbon receptor, KYN = kynurenine, KYNA = kynurenic acid, IDO1 = indoleamine 2,3-dioxygenase 1, TDO2 = tryptophan 2,3-dioxygenase, Trp = Tryptophan, TAM = tumor-associated macrophages.

PC often goes undetected until it reaches an advanced stage, making it difficult to undergo radical resection and resulting in a poor prognosis. Even for resected patients, the recurrence rate remains high, seriously affecting the 5-year survival rate for PC patients. Unresectable PC patients do not receive traditional chemotherapy based on fluorouracil or gemcitabine as a standard treatment. However, some studies have shown that the advancement of immunotherapy, especially immune checkpoint blockade therapy, has achieved significant results in solid tumors.^[19–21]^ As illustrated in Figure 2, the difference between normal metabolism and PC metabolism is mainly reflected in the overexpression of IDO1 and the depletion of Trp, leading to elevated levels of L-KYN and sustained activation of AhR, thereby promoting tumor growth and the formation of immune tolerance. Therefore, Trp metabolism and its products can be an immune regulatory factor to improve the immune microenvironment. In 2023, Yang and colleagues confirmed that the excessive expression of IDO1 inhibits the maturation of dendritic cells in the liver and spleen. This results in reduced expression of natural killer T cells and can cause an imbalance in the ratio of T helper 17 cells (Th17 cells)/Treg cells.^[22]^ Therefore, Trp metabolites are a promising treatment target for PC patients tolerant to immunotherapy. Anu found that IDO1 and its metabolite KYN play a crucial role in the immune microenvironment, regulating the immune system response. Additionally, KYN suppresses T cell function through various mechanisms, including inhibition of T cell apoptosis, induction of T cell proliferation, and suppression of T cell survival. For example, the IDO1-KYN-NAD^+^ pathway produces immunosuppressive metabolites to inhibit the function of immune cells, thereby promoting the immune escape of tumors.^[23]^ Sun found the critical process and molecular mechanism of tumor-associated nonmyelinating Schwann cells in PDAC. Plasmacytoma variant translocation 1 in tumor-associated nonmyelinating Schwann cells promotes PC tumor occurrence and inhibits cytotoxic CD8^+^ T cells presence in the tumor microenvironment by regulating TDO2 phosphorylation.^[24]^

### 3.3. Effects of tryptophan metabolites on metastasis and proliferation of pancreatic cancer

The activation of the Trp metabolism pathway is linked to the increased migration, proliferation, and invasion capability of PC cells. Metabolites such as KYNA and 5-HT can act as promoting factors for tumor growth and metastasis. In 2018, Saponara found that overexpression of 5-HT promotes the migration and invasion of PC cells, while the use of SSRIs drugs to inhibit 5-HT uptake can reduce the proliferation and migration of PC cells.^[25]^ In 2020, Wang studied the effect of KYNA on cell growth using Capan-1 PC cell spheroids. They grew the cells in 3D culture and treated them with 10, 50, and 100 μM doses of KYNA. The study found that compared to the control group, treatment with KYNA led to enhanced spheroid growth in a dose-dependent manner, demonstrating that KYN promoted the mechanism of proliferation and metastasis of PC cell spheroids.^[26]^ Therefore, Trp can promote PC cell proliferation and migration via 5-HT and KYN ways. This promotes tumor progression by inhibiting anti-tumor immune responses and increasing cancer cell malignant characteristics.

### 3.4. Drug of tryptophan metabolism affects the progression of pancreatic cancer

Pharmaceutical interventions targeting the Trp metabolism pathway, such as IDO1 inhibitors, have shown potential value in PC treatment by regulating the tumor immune microenvironment and enhancing patient response to existing treatments. Upregulation of the rate-limiting enzyme IDO1 promotes the conversion of Trp to KYN, thereby strengthening the presence of Treg and M2 tumor-associated macrophages in the tumor microenvironment.^[27]^ In cancer cells, increased Trp degradation releases immune-suppressive metabolic byproducts that upregulate the PD-1 co-stimulatory molecule on CD8^+^ T cells.^[27,28]^ Alleviating the immune-suppressive microenvironment of PC can be achieved by inhibiting the rate-limiting enzyme IDO1.^[29]^

On the other hand, the gut microbiota plays a vital role in the metabolism of Trp degradation, leading to immune suppression and tumor growth in pancreatic malignancy. Research has indicated that enhancing the efficacy of chemotherapy in a humanized germ-free mouse model of PDAC can be achieved by short-term dietary control of Trp, fecal microbiota transplantation, and oral administration of drugs identified by 3-IAA. Studies using knockout and overexpression have demonstrated that the efficacy of 3-IAA drugs and chemotherapy is mediated through neutrophil-derived myeloperoxidase. Myeloperoxidase is an enzyme that oxidizes a specific substance. Combined with chemotherapy, it leads to the downregulation of enzymes (glutathione peroxidase-3 and glutathione peroxidase-7) that break down reactive oxygen species. This results in the accumulation of ROS within cancer cells, which reduces their ability to undergo autophagy (a process of self-degradation). As a result, cancer cell metabolism and proliferation are decreased. In 2 independent cohorts of PC patients, clinical trials demonstrated a significant correlation between treatment effectiveness and 3-IAA levels.^[30]^

In the field of nanomedicine, Huang found that oxaliplatin combined with liposome-encapsulated IDO1 siRNA nanodrug delivery to tumor tissue and tumor-draining lymph nodes can promote dendritic cell maturation, increase intratumoral T lymphocyte infiltration in PC, reduce Treg cells, and provide a potent immune memory effect, achieving synergistic anti-tumor effects.^[31]^

In traditional Chinese medicine, it is believed that an excess of “dampness, heat, and toxins” plays a vital role in the development of PC through causing spleen dampness and qi stagnation. The progression of the disease is marked by distinct pathological alterations in the accessory organs, leading to atypical pancreatic fluid secretion and excretion. This culminates in the buildup of stagnant fluids and unhealthy dietary practices, which ultimately pave the way for the onset of cancer.^[32]^ Triptolide is the primary active compound of *Tripterygium wilfordii*. It has many functions such as pain relief, detoxification, and improving blood flow.^[33]^ In 2021, Lou designed and synthesized a Trp-conjugated triptolide prodrug to target the high expression of amino acid transporter B⁰,^+^(ATB^0^, ^+^) in PC cells, thereby more effectively treating pancreatic malignancy.^[34]^

### 3.5. Intestinal flora regulates tryptophan metabolism and affects the progression of pancreatic cancer

Some studies indicated that the diversity and alterations of the intestinal microbiota may be associated with initiation and progression of PDAC.^[35,36]^ In a recent study, researchers collected and analyzed fecal samples from 57 healthy controls and 85 PDAC patients for microbial features. The results showed that PDAC patients had significantly lower gut microbiota diversity than the control group. Compared to the healthy control group, patients with PDAC showed a significant increase in the abundance of Fusobacteria and a decrease in the quantity of Firmicutes and Bacteroidetes phyla.^[37]^ In 2020, Kohi conducted a study on the duodenal fluid fungal and bacterial diversity of 74 PDAC patients, 98 patients with pancreatic cystic neoplasms, and 134 normal individuals. The results showed that there was a significant reduction in both fungal and bacterial diversity among PDAC patients as compared to the other 2 groups.^[38]^ There was no significant difference in the duodenal microbiota of patients with pancreatic cystic neoplasms and healthy individuals. Additionally, Professor Tracy L. McGaha’s team explored the impact of AhR^+^ activation in tumor-associated macrophages on the immune response in PDAC. According to the study, macrophages lacking AhR gene developed an inflammatory phenotype. Inhibiting AhR could reduce PDAC growth, enhance the effectiveness of immune checkpoint blockade, and increase the frequency of IFNγ^+^CD8^+^ T cells within the tumor. Therefore, the study demonstrated that diet metabolizes Trp through the lactobacilli group, producing indole compounds that drive AhR, activating the function of tumor-associated macrophages.^[28]^

PC patients may impact gut microbiota composition, influencing Trp metabolism, short-chain fatty acid production, and immune system function. Short-chain fatty acids are crucial for regulating immune tolerance, improving intestinal barrier integrity, and clearing the gut. Ensuring sufficient levels of short-chain fatty acids is vital for maintaining a healthy metabolism and mitigating the risk of various diseases.^[39,40]^ In a recent study, it was observed that mice subjected to a low-fiber diet experienced a reduction in butyrate secretion, which in turn directly impacted the diversity of gut microbiota. This led to systemic inflammation and necrotizing pancreatitis, ultimately culminating in mortality. In patients with late-stage PDAC, there exists a unique relationship between the gut microbiome and enteral nutrition therapy for cancer cachexia. Modulating the fecal microbiome could be an effective strategy for alleviating cachexia associated with PDAC.^[41]^

## 4. Conclusion and future perspective

Trp is mainly metabolized through the KYN, 5-HT, and indole pathways. The microbiota residing in the gastrointestinal system can exert a direct or indirect influence on the metabolism of Trp through the production of metabolites. IDO1 and TDO2 are enzymes that limit the rate of Trp metabolism. Significant progress has been made in developing inhibitors for these enzymes. Understanding Trp metabolism is vital in the development of pancreatic malignancy. Its metabolites have potential medical applications in diagnosis, prognosis assessment, and treatment strategies. In conclusion, there is a need for further research to investigate the exact molecular mechanisms that connect Trp metabolism and pancreatic malignancy, as well as how diet controls the gut microbiota to regulate Trp metabolism pathways affecting the progression of PC, providing new strategies for the early diagnosis and treatment of PC.

## Acknowledgments

This work was carried out by Lei Yang (Tianjin NanKai Hospital; Tianjin Key Laboratory of Acute Abdomen Disease Associated Organ Injury and ITCWM Repair; Institute of Integrative Medicine for Acute Abdominal Diseases). We gratefully acknowledge his invaluable cooperation in preparing this study.

## Author contributions

**Conceptualization:** Lei Yang, Ximo Wang.

**Formal analysis:** Yifei Gao.

**Writing – original draft:** Yifei Gao.

**Writing – review and editing:** Ximo Wang.
